# Effects of fertilizer under different dripline spacings on summer maize in northern China

**DOI:** 10.1038/s41598-021-98016-6

**Published:** 2021-09-23

**Authors:** Ge Li, Youlu Bai, Lei Wang, Yanli Lu, Jingjing Zhang, Yinjie Zhang

**Affiliations:** 1 Key Laboratory of Plant Nutrition and Fertilizer, Ministry of Agriculture and Rural Affairs/ Institute of Agricultural Resources and Regional Planning, Chinese Academy of Agricultural Sciences, 12 South Road, Zhongguancun, Haidian District, Beijing, 100081 People’s Republic of China; 2 Institute of Environment and Sustainable Development in Agriculture, Chinese Academy of Agricultural Sciences, Beijing, 100081 People’s Republic of China

**Keywords:** Plant sciences, Ecology, Sustainability

## Abstract

Maximizing grain yields with effective fertilization technologies and minimizing nitrogen losses is essential in agroecosystems. In this research, we conducted a two-year field experiment to explore whether dripline spacing and fertilization rate would affect maize grain yield. Two dripline spacings (i.e., one drip line per row of maize with a row space of 60 cm and one drip line per two rows of maize) and two fertilization rates (i.e., high fertilization level: N, 180 kg ha^−1^; P_2_O_5_, 90 kg ha^−1^; and K_2_O, 90 kg ha^−1^ and low level: N, 139.5 kg ha^−1^; P_2_O_5_, 76.5 kg ha^−1^; and K_2_O, 76.5 kg ha^−1^) were employed in this research. The results showed that maize yield was significantly affected by both dripline spacing and fertilization rate. The maize yield was 10.2% higher in the treatment with one drip line per two rows than that in the treatment with one drip line per row. Maize yield increased by 10.9% at the high fertilization level compared to that at the low fertilization level. The quantity of cumulative ammonia volatilization was reduced by 15.1% with one drip line per two rows compared to that with one drip line per row, whereas it increased by 26.9% at the high fertilization level compared with that at the low fertilization level. These results indicated that one drip line per two rows with a high fertilization rate increased the yield and could reduce the environmental burden, which may be economically beneficial and environmentally sound for maize fertigation for green agricultural development.

## Introduction

As an important food crop in China, the planting area of maize (*Zea mays* L.) accounts for 25.4% of the total planting area of crops^1^, and maize production has a direct impact on national food security and the development of the grain industry^2^. The application of chemical fertilizers, especially nitrogen (N), has made tremendous contributions to improving grain yields (GYs) and food security in China. The high input and high output production system in China heavily relies on the use of chemical fertilizers, especially N fertilizer, which results in high N emissions and low N use efficiency (NUE) in intensive cropping systems^3^. The North China Plain is one of the major maize-producing regions; however, the total ammonia volatilization losses were shown to be over 3 million t yr^−1^, which has been identified as a global hotspot for ammonia^4,5^. There is an urgent need for high-efficiency fertilization methods for summer maize cultivation in this region.

At present, the fertigation method using drip fertigation systems has been widely used internationally in modern agriculture due to its large irrigation area coverage, high fertilizer utilization efficiency, high degree of automation, low labour demand, and low environmental impacts^6–10^. Maize crops under drip irrigation conditions with N fertilization have been widely tested and have shown successful results, exhibiting increased GY and economic benefits, improved utilization efficiency of fertilizers and water, and reduced N loss^7,11–13^. However, critical management considerations such as dripline spacing and fertilization rate are necessary to attain improved crop productivity, nutrient and water use efficiency, and production benefits. Initially, the research on dripline spacing was conducted to reduce investment costs to promote drip irrigation technology^14^, and then Bozkurt et al.^15^ found that changes in dripline spacing resulted in significantly different yield. Chen et al.^16^ claimed that dripline spacing affected the leaf area index, net photosynthetic rate and aboveground biomass, and plant growth and GY both decreased as dripline spacing increased. Zhou et al.^17^ evaluated the effects of dripline spacing on the distributions of soil water and nitrate and found that narrower dripline spacing could enhance the distribution uniformity of NO_3_-N concentration, relative chlorophyll content of leaves, and crop yield. However, the relationship between dripline spacing and crop yield cannot be considered universal, as the efficiency of fertigation varies greatly in fields with different agricultural measures (fertilizer type, fertilization and irrigation rate), soil and climatic factors^18–20^. Appropriate fertilizer management needs to consider the specific relations between N fertilization rates, growing season characteristics, and soil texture in maize production^21^. Nitrogen fertilization affected the grain-filling process for the achievement of high GY^22^, and excess or insufficient fertilization was not conducive to the yield^23^. However, there has been insufficient research on the interaction between dripline spacing and fertilization rate for maize production in northern China. To achieve high crop production and sustainable agricultural development, it is necessary to compare the effects of different dripline spacings and fertilization rates on maize yield.

Improving N fertilizer management and reducing ammonia loss are crucial for improving maize yield and reducing adverse environmental impacts. Soil ammonia volatilization is decisively influenced by many factors, including climatic conditions, soil properties, the application amount of N and the fertilization methods^24^. Reducing the amount of N fertilizer use can decrease ammonia volatilization, but the space for reducing the amount of fertilizer is limited to maintain crop yield^25^. Some studies reported that fertilization methods (combined with biochar, straw return, deep fertigation, film mulching) and modified fertilizer can mitigate ammonia volatilization and increase NUE in agroecosystems^26–30^, whereas other results found that these measures have been limited generalization due to the complex production process, lack of proper operating machines, and uncertain application effects^27,31–34^. However, it has been recognized that fertigation is an efficient strategy for controlling the placement, time, and N fertilization rate, thereby increasing NUE^35^. A 4-year study of a tea plantation indicated that drip fertigation is a good management practice that not only reduces total N and total phosphorous losses to environment but also sustains yield^36^. Some studies have reported that the appropriate fertigation method may minimize levels of ammonia volatilization, N_2_O emissions and nitrate leaching in plant-soil-atmosphere systems^37,38^. Nonetheless, few measurements of ammonia volatilization under drip fertigation from applied fertilizer in northern China have been reported, and ammonia loss under different dripline spacings and fertilization rates has not yet been evaluated in this region.

Spatial variability is one of the most interesting factors impacting fertilization management optimization and monitoring of the evolution of soil functions^39^. Soil nutrients have complex scale-dependent interrelations and spatial variability. Studies have generally focused on the large-scale (such as grass, forestland and farmland) spatial distribution of soil nutrients^40,41^, but the small-scale spatial distribution of soil nutrients also needs attention to serve as a basis for fertigation and soil management of maize fields. However, little information is available on the spatial variability in soil properties between dripline spacing and fertilization rate. Understanding the effects of fertilizer under different dripline spacings on maize is urgently needed to provide a scientific foundation and theoretical basis for achieving efficient fertigation methods and reduced environmental risk.

The aim of this research was to (a) evaluate the effect of different dripline spacings and fertilization rates on the yield of summer maize, (b) clarify the effect of dripline spacings and fertilization rates on ammonia emissions, (c) reveal the spatial variability in soil nutrients under fertigation, and (d) assess effective strategies to mitigate ammonia losses and increase crop yield in northern China.

## Materials and methods

### Experimental site description

**Figure 1:**
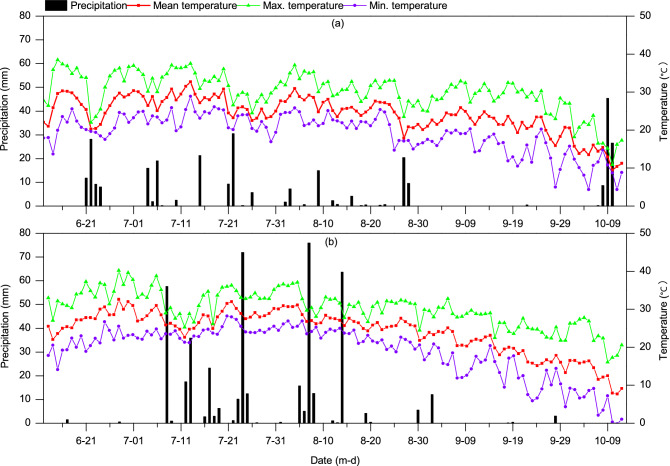
Daily precipitation and temperature of the experimental field during the summer maize growing seasons in 2017 (**a**) and 2018 (**b**). Graphs were created using Origin software version 2021 (Origin Lab Ltd., Guangzhou, China). Origin is used herein under license. Copyright OriginLab Corporation. All rights reserved. For more information about Origin software, please visit www.originlab.com.

Field experiments were carried out from June to October in 2017 and 2018 at the International Agricultural Emerging Industrial Park of the Chinese Academy of Agricultural Sciences (39° 35′N, 116° 35′E) located in Langfang, Hebei Province, China. The climate is a typical temperate continental monsoon climate. The long-term (1981–2010) annual mean air temperature, precipitation and hours of sunshine were 11.8 °C, 503.4 mm and 2487.2 h, respectively. Figure 1 shows the daily air temperature and precipitation during the maize growing seasons in 2017 and 2018. The soil is a sandy loam, and the farming system in the study site is winter wheat-summer maize double cropping. The chemical properties of the soil of the experimental field (Table 1) were determined by the National Soil Testing and Fertilization Center using ASI methods^42^.

**Table 1:** Basic chemical properties of the soil at experimental site in 2017.

Depth (cm)	NO_3_^−^-N (mg L^−1^)	NH_4_^+^-N (mg L^−1^)	Available phosphorus (mg L^−1^)	Available potassium (mg L^−1^)	Organic matter (%)	pH	Available sulfur (mg L^−1^)
0–20	21.6	1.8	17.0	74.0	0.66	8.73	15.2
20–40	21.5	0.9	6.5	50.3	0.49	8.72	21.5

Depth (cm)	Available boron (mg L^−1^)	Available iron (mg L^−1^)	Available manganese (mg L^−1^)	Available copper (mg L^−1^)	Available zinc (mg L^−1^)	Available calcium (mg L^−1^)	Available magnesium (mg L^−1^)
0–20	2.6	10.8	16.5	0.3	0.6	1521.1	241.5
20–40	2.4	9.3	16.5	0.3	0.5	1662.1	231.2

### Experimental design and fertigation system

**Figure 2:**
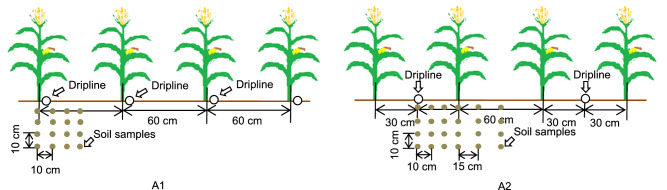
Schematic diagram of layout of one drip line per row of maize (A1), one drip line per two rows of maize (A2) and soil samples. Graphs were created using Microsoft Office version 2010. Microsoft Office is used herein under license. Copyright Microsoft Corporation. All rights reserved. For more information about Microsoft Office, please visit www.microsoft.com.

“Zhengdan958”, a maize (*Zea mays* L.) cultivar widely grown in the experimental area, was used for this study. To evaluate the effects of fertilizer under different dripline spacings on summer maize, a two-factor randomized block design with two replications (because the plot size was larger than the usual test plot) was adopted, which compared two dripline spacings and two fertilization levels over the two growing seasons. The dripline spacing was one dripline per row of maize (treatment A1) and one dripline per two rows of maize (treatment A2). The drip spacing was 0.6 m in treatment A1 and 1.2 m in treatment A2 (Fig. 2). The drip fertigation belt was patch-type with a dripper spacing of 0.1 m and a dripper flow rate of 2 L h^−1^. The two fertilization levels (Table 2) were 180 kg ha^−1^ N, 90 kg ha^−1^ P_2_O_5_, and 90 kg ha^−1^ K_2_O (treatment F1) and 139.5 kg ha^−1^ N, 76.5 kg ha^−1^ P_2_O_5_, and 76.5 kg ha^−1^ K_2_O (treatment F2), with nutrient (N, P_2_O_5_ and K_2_O) application rate of drip-fertigation in treatment F2 as 70% of that in treatment F1. In both fertilization treatments, the same rate of basal fertilizer was applied with seeding at 45 kg ha^−1^ for each of the three nutrients (N, P_2_O_5_ and K_2_O) (Table 2). The basal fertilizer was 15–15–15 (N-P_2_O_5_-K_2_O) compound fertilizer. For drip fertigation, N fertilizer was applied in equal thirds at the seven-leaf stage (V7), ten-leaf stage (V10) and silking stage (R1); the phosphate fertilizer was applied at the V7 stage, while potassium (K) fertilizer was applied at a ratio of 2:1 at the V7 stage and R1 stage (Table 3). The three fertilizers were applied by the drip fertigation system in the field experiment. A mechanized seeder was used to sow seeds and apply the seed fertilizer simultaneously. The plot size was 1300 m^2^ (65 m in length and 20 m in width) for each treatment. Maize was planted with a row spacing of 60 cm and plant spacing of 25 cm. The maize was sown on June 12 in 2017 and June 17 in 2018. Harvest dates were October 12 in 2017 and October 10 in 2018. Other field management procedures, including weeding, pest control and chemical control, were the same for all treatments.

**Table 2:** Seed, drip and total fertilization rates in the field experiment.

Treatment	Seed-fertilization rate (kg ha^−1^)			Drip-fertigation rate (kg ha^−1^)			Total fertilization rate (kg ha^−1^)		
	N	P_2_O_5_	K_2_O	N	P_2_O_5_	K_2_O	N	P_2_O_5_	K_2_O
F1^a^	45	45	45	135	45	45	180	90	90
F2	45	45	45	94.5	31.5	31.5	139.5	76.5	76.5

^a^F1 and F2 are the high and low fertilization rate, respectively.

**Table 3:** Fertigation scheduling, precipitation and irrigation amount during summer maize growing seasons.

Treatment			F1^a^	F2
Drip-fertigation amount (L plot^−1^)	N barrel	The seven-leaf stage	101.7	71.2
		The ten-leaf stage	127.2	89.0
		The silking stage	127.2	89.0
	P barrel	The seven-leaf stage	195.0	136.5
	K barrel	The seven-leaf stage	125.8	88.1
		The silking stage	62.9	44.0
Precipitation (mm)	2017	June 12-October 12	306.5	306.5
	2018	June 17-October 10	444.2	444.2
Irrigation (mm)	2017	June 12-October 12	84.0	84.0
	2018	June 17-October 10	70.5	70.5

^a^F1 and F2 are the high and low fertilization rate, respectively.

**Figure 3:**
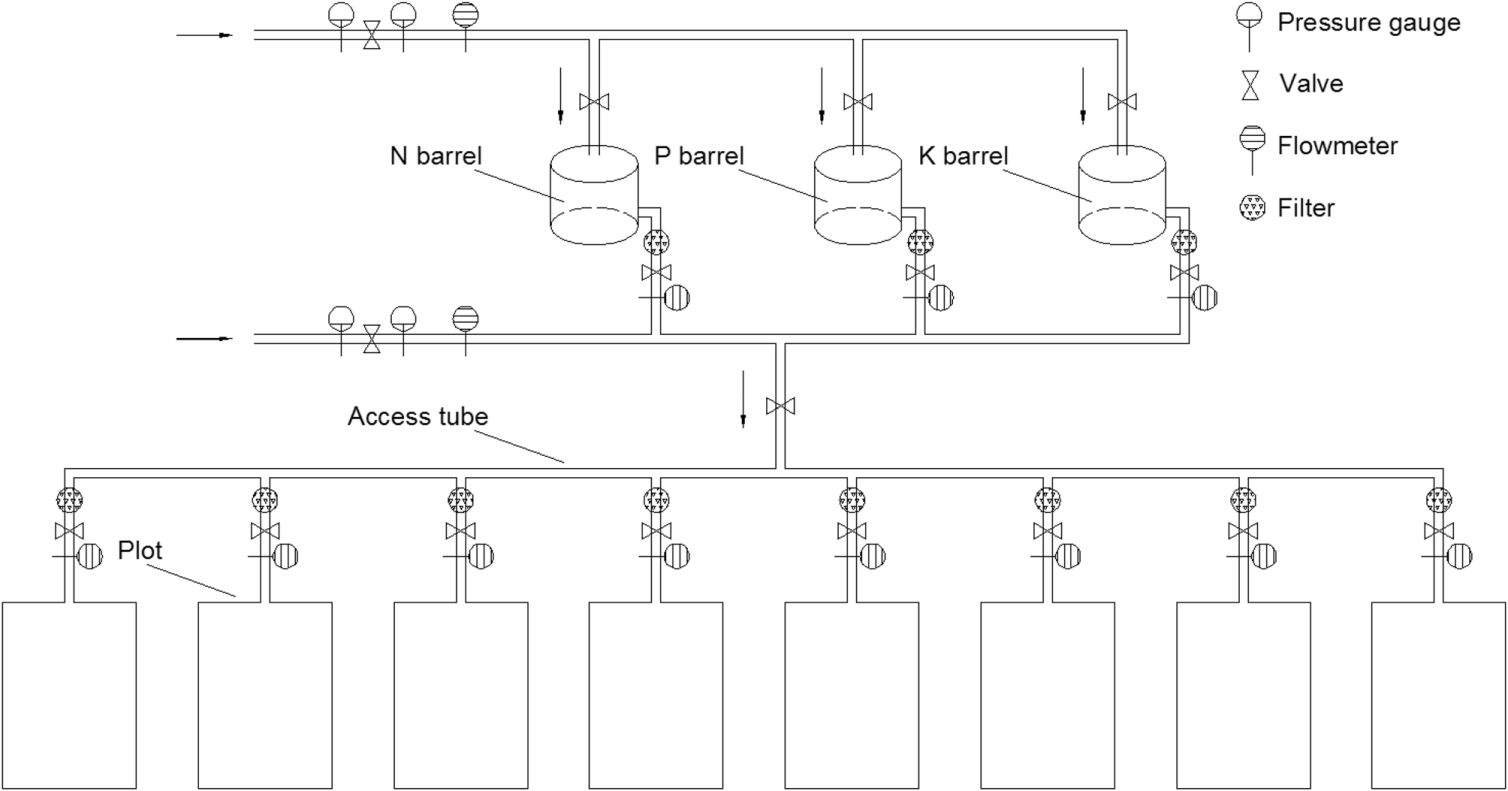
Schematic diagram of the drip fertigation system. Graph was created using AutoCAD software version 2007 (Autodesk, USA). AutoCAD is the intellectual property of Autodesk and is used herein under license. Copyright Autodesk Corporation. All rights reserved. For more information about AutoCAD software, please visit www.autodesk.com.

The drip fertigation system that for application of fertilizer with water consisted of three fertilizer storage barrels with a volume of 1000 L, several piston injection pumps, a number of solenoid valves, multiple flow meters and filters. All circuits were integrated on a circuit board and controlled by computer programs (Fig. 3). The N liquid storage configuration was as follows: 100 kg of urea (46% N) was dissolved in a 1000 L N barrel to prepare a fertilizer stock solution of 0.046 kg N L^−1^. The phosphate liquid storage configuration was as follows: 50 kg of ammonium dihydrogen phosphate (12% N, 60% P_2_O_5_) was dissolved in a 1000 L phosphorus (P) barrel to prepare a fertilizer stock solution of 0.006 kg N L^−1^ and 0.03 kg P_2_O_5_ L^−1^. The K liquid storage configuration was as follows: 50 kg of potassium chloride (62% K_2_O) was dissolved in a 1000 L K barrel to prepare a fertilizer stock solution of 0.031 kg K_2_O L^−1^. The amount of N, P and K fertilizer stock solution liquid and irrigation water for every plot was independently controlled by solenoid valves through a computer operating system. All plots received the same irrigation amount during the whole growth period of maize.

### Measurements and calculation

#### GY and yield components

All plants in each plot were harvested after crop maturation. Air-dried GY (kg ha^−1^) was obtained in each plot and weighed^43^, and ear length (EL), ear diameter (ED), kernel number per ear (KN), and 100-kernel weight (KW) were determined.

#### Measurement of dry matter yield (DM) and nutrient uptake

Six plants were randomly sampled at harvest from each plot and separated into leaves, stalks and grains. These tissues were oven-dried at 105 °C for 30 min and then dried to constant weight at 75 °C. After measuring DM, the plants were ground, and a subsample was digested in concentrated H_2_SO_4_-H_2_O_2_ for the determination of N, P and K concentrations. Total N and total P were determined by colorimetry using a flow analyser (Seal AA3, Germany), and total K was determined by atomic absorption spectrometry (AAnalyst 100, USA). Total plant N, P and K uptake in the aboveground part of maize plants was calculated based on plant DM and N, P and K concentrations, respectively^44^.

#### Ammonia emissions

Ammonia volatilization was determined by the ventilation method^45^. The measuring device comprised a polyvinyl chloride rigid plastic tube with an outer diameter of 16 cm and a height of 10 cm^46^. Six measuring devices were evenly placed beside the drip irrigation belt in each treatment. Ammonia volatilization measurement started from the day fertigation began at the V7 stage and ended the day before fertigation at the V10 stage. Samples were taken at 7:30 am, and the upper sponge was replaced every 3 days to prevent other gases in the external environment from disturbing the determination of ammonia volatilization. The lower sponge was replaced every day and put into a marked valve bag. The sponge was returned to the laboratory and placed in a 500 mL plastic bottle to which 300 mL of 1.0 mol L^−1^ KCl solution was added, and the bottle was then shaken for 1 h. Finally, the ammonium N content in the extract was determined by colorimetry using a flow analyser (Seal AA3, Germany). The ammonia volatilization rate (kg ha^−1^ day^−1^) was calculated following Eq. (1), and cumulative ammonia volatilization (kg ha^−1^) was the sum of the ammonia volatilization rate.

1 $$Ammonia\,volatilization\,rate = M/\left( {A \times T} \right) \times 10^{ - 2}$$

where *M* is the amount of ammonia measured in a measuring device (mg), *A* is the cross-sectional area of the capture device (m^2^), and *T* is the time of each continuous collection (d).

#### Soil samples

Destructive soil samples were collected from each plot with an auger at harvest in the 2017 maize season. The distances to the drip laterals for sampling were 0, 10, 20, 30 (A1 horizontal sampling to 30 cm), 45, and 60 (A2 horizontal sampling to 60 cm) cm, and samples were taken from the 0–10, 10–20, 20–30, and 30–40 cm layers. The layout of the drip laterals and positions of the soil samples are shown in Fig. 2. In total, from the horizontal and vertical directions, sixteen soil samples were collected in the A1 treatment group, and twenty-four soil samples were collected in the A2 treatment group. The soil sample was air-dried and passed through a 1 mm sieve; mineral N was extracted with 2.0 mol L^−1^ KCl, filtered through filter papers, and then subjected to colorimetric determination of ammonium-N content and nitrate–N content in a flow analyser (Seal AA3, Germany). Available P was extracted with 0.5 mol L^−1^ NaHCO_3_ solution, and the molybdenum antimony colorimetric method was used to determine soil available P content; available K was extracted with 1.0 mol L^−1^ ammonium acetate solution and determined by atomic absorption method (AAnalyst 100, USA)^47^. The coefficient of variation (CV) was calculated according to Eq. (2), and the Christiansen uniformity coefficient (CU) was calculated according to Eq. (3)^48^.

2 $$CV = \frac{SD}{{\overline{x}}}\times 100\%$$

where *SD* is the standard deviation of soil nutrient content and $\overline{x}$ is the average soil nutrient content.

3 $$CU = \left( {1 - \frac{{\mathop \sum \nolimits_{i = 1}^{N} \left| {xi - \overline{x}} \right|}}{{N\overline{x}}}} \right) \times 100\%$$

where *x*_*i*_ is the soil nutrient content, $\overline{x}$ is the average value of *x*_*i*_, and *N* is the number of soil samples.

### Statistical analysis

All of the experimental data were processed using Excel 2010 (Microsoft Office, USA). The data were subjected to analysis of variance (ANOVA) by SAS software version 9.2 (SAS Institute, Cary, NC), and multiple comparisons were performed using Duncan’s multiple-range test (*P* < 0.05), unless otherwise stated. Pairwise Pearson correlation significance was used to determine the relationship between GY, yield components and DM. Figure 2 was drawn with Word 2010 (Microsoft Office, USA), Fig. 3 was drawn with AutoCAD software version 2007 (Autodesk, USA), and the other graphs were prepared with Origin software version 2021 (Origin Lab Ltd., Guangzhou, China).

### Statement

“Zhengdan958”, the maize (*Z. mays* L.) cultivar that we used in the present experiment, complied with international guidelines. We complied with the IUCN Policy Statement on Research Involving Species at risk of extinction and the Convention on the Trade in Endangered Species of Wild Fauna and Flora.

## Results

### GY and yield components

ANOVA showed that the impact of dripline spacing on GY in 2017 was significant (*P* < 0.01), with GY being 12.5% greater in the treatment with one drip line per two rows than in the treatment with one drip line per row (Table 4). The effect of fertilization rate on maize yield was also significant (*P* < 0.05), with GY in the high fertilization treatment exceeding that in the low fertilization treatment by 9.8%. The interactive effect on maize yield between dripline spacing and fertilization rate was not significant (*P* > 0.05). There was no significant (*P* > 0.05) difference in EL between the two drip line treatments; however, ED and KW were significantly (*P* < 0.05) greater in the one drip line per two rows treatment than in one drip line per row treatment. Fertilization rate had no significant (*P* > 0.05) effect on the EL, ED, or KW. However, there was a significant (*P* < 0.01) interactive effect between dripline spacing and fertilization rate on the KN. The KN at the low fertilization rate in the one drip line per row treatment was significantly (*P* < 0.05) lower than that with the same fertilization rate but in the one drip line per two rows treatment.

**Table 4:** The grain yield and yield components of summer maize under different treatments in 2017 and 2018.

Year	Treatment^a^	Ear length (cm)	Ear diameter (cm)	Kernel number per ear	100-kernel weight (g)	Grain yield (kg ha^−1^)
2017	A1F1	17.83a^b^	5.11ab	553.75b	34.24a	7536.54bc
	A1F2	17.88a	5.01b	504.27c	33.43a	6925.00c
	A2F1	18.13a	5.25a	569.84a	37.97a	8546.15a
	A2F2	17.56a	5.21a	559.17ab	37.51a	7717.31b
2018	A1F1	16.40a	4.81b	474.33b	31.40b	6048.79ab
	A1F2	15.88ab	4.82b	490.95b	30.38b	5370.81c
	A2F1	16.31a	4.97ab	543.00a	34.45a	6494.89a
	A2F2	15.53b	5.02a	466.11b	33.82a	5833.71bc
2017	Source of variance	*F* value	*F* value	*F* value	*F* value	*F* value
	A	0.00	14.01**	58.89**	6.34*	28.98**
	F	0.29	2.41	42.27**	0.17	18.52*
	A × F	0.39	0.41	17.61**	0.01	0.42
2018	Source of variance	*F* value	*F* value	*F* value	*F* value	*F* value
	A	0.93	13.30**	6.49*	43.37**	8.60*
	F	7.77*	0.35	12.27**	2.76	18.67*
	A × F	0.33	0.18	29.54**	0.16	0.00

^a^A and F represent dripline spacing and fertilization rate, respectively, as shown in Fig. 2 and Table 2.

^b^Values within a column followed by different letters are significantly different at the 0.05 probability level; * and ** show significant difference at the 0.05 and 0.01 probability levels, respectively.

In 2018, GY was significantly (*P* < 0.05) affected by dripline spacing treatment, with GY in the one drip line per two rows treatment being 8.0% higher than that in the one drip line per row treatment (Table 4). Fertilization rate also significantly (*P* < 0.05) affected GY, with the yield in the high fertilization rate treatment being 12.0% higher than that in the low fertilization rate treatment. No interactive effect between dripline spacing and fertilization rate on maize yield was found. Dripline spacing significantly (*P* < 0.01) affected maize ED and KW, with one drip line per two rows showing better results than one drip line per row. However, the dripline spacing did not significantly (*P* > 0.05) influence the maize EL in this study. Fertilization rate did not significantly (*P* > 0.05) affect maize ED or KW, but the EL was reduced at the low fertilization rate. There was a similar interactive effect between dripline spacing and fertilization rate on the KN in 2018 and 2017.

### DM and nutrient uptake

**Figure 4:**
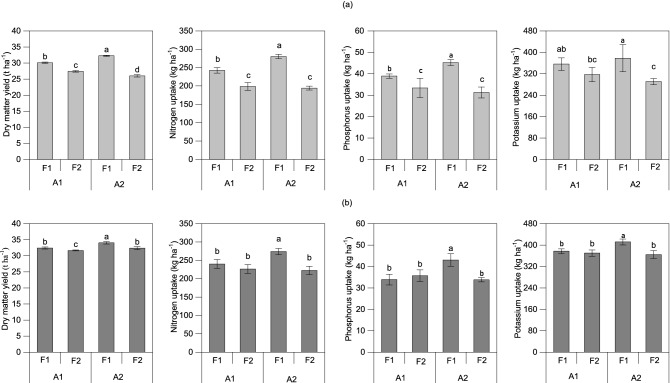
Effects of dripline spacing and fertilization rate on dry matter yield and nutrient uptake of summer maize in 2017 (**a**) and 2018 (**b**). Values within a column followed by different letters are significantly different at the 0.05 probability level. Error bars show ± standard deviation. A1, A2, and F1, F2 represent dripline spacing and fertilization rate, respectively, as shown in Fig. 2 and Table 2. Graphs were created using Origin software version 2021 (Origin Lab Ltd., Guangzhou, China). Origin is used herein under license. Copyright OriginLab Corporation. All rights reserved. For more information about Origin software, please visit www.originlab.com.

In 2017, there was a highly significant (*P* < 0.01) interactive effect between dripline spacing and fertilization rate on DM (Table 5). DM was significantly (*P* < 0.01) higher at the high fertilization rate than at the low rate, and the difference was larger where there was one drip line per two rows than where there was one drip line per row. At the high fertilization rate, the DM of maize plants was higher in the one drip line per two rows treatment than in the one drip line per row treatment. The total uptake of N, P and K in the aboveground part of the plant was significantly higher at the high fertilization rate than at the low fertilization rate (Fig. 4). As with DM, the effect of dripline spacing on the total uptake of N and P by the plant aboveground parts highly depended on the fertilization rate. The cumulative uptake of N and P was significantly (*P* < 0.05) greater in the one drip line per two rows of treatments than in the one line per row of treatments at the high fertilization rate but not at the low P rate. No significant effect of dripline spacing on the total uptake of K by plants was found.

**Table 5:** Effects of dripline spacing and fertilization rate on dry matter yield and nutrient uptake of two-way analysis of variance (*F* value).

Year	Source of variance^a^	Dry matter yield	Nitrogen uptake	Phosphorus uptake	Potassium uptake
2017	A	6.58*^b^	14.03**	1.72	0.01
	F	924.27**	215.09**	38.12**	12.02**
	A × F	149.56**	22.55**	7.19*	1.71
2018	A	34.74**	5.49*	7.01*	4.57
	F	37.54**	25.52**	7.22*	16.14**
	A × F	5.22	8.49*	16.17**	8.85*

^a^A and F represent dripline spacing and fertilization rate, respectively, as shown in Fig. 2 and Table 2.

^b^* and ** show significant difference at the 0.05 and 0.01 probability levels, respectively.

The results from the 2018 experiment indicated that there was no significant (*P* > 0.05) interactive effect between dripline spacing and fertilization rate on DM (Table 5). DM was significantly (*P* < 0.01) higher at the high fertilization rate than at the low fertilization rate, and it was significantly (*P* < 0.01) higher in the one drip line per two rows treatment than in the one drip line per row treatment. At the high fertilization rate, DM was greater in the one drip line per two rows of treatment in 2018, as was found in 2017. The total uptake of N, P and K was significantly (*P* < 0.05) higher at the high fertilization rate than at the low fertilization rate. This effect of fertilization rate on nutrient uptake became greater with one drip line per two rows than with one drip line per row. The effect of dripline spacing on N uptake was less than the fertilization rate effect. The cumulative uptake of N, P and K was greater in the one drip line per two rows treatment than in the one line per row treatment at the high fertilization rate. No significant effect of dripline spacing on the total uptake of K was found.

### Correlation analysis of GY, yield components and DM

**Figure 5:**
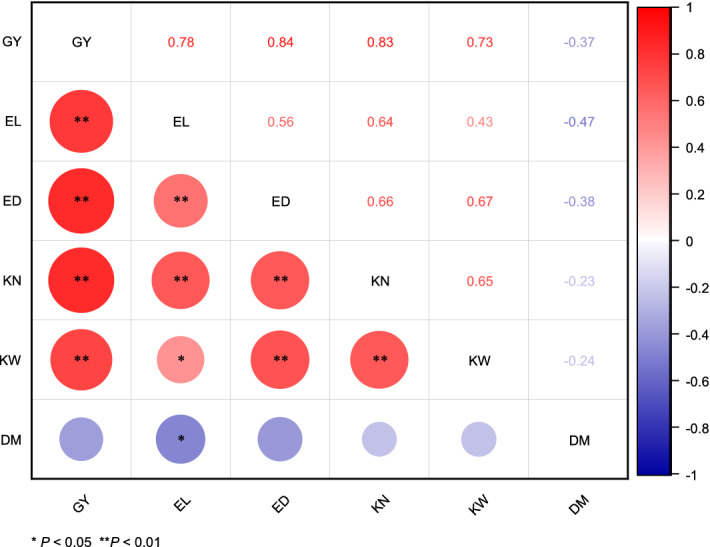
Correlation analysis of grain yield (GY), ear length (EL), ear diameter (ED), kernel number per ear (KN), 100-kernel weight (KW), and dry matter yield (DM). Correlation values show in the upper triangular, significant mark show in the lower triangular, and * and ** show significant difference at the 0.05 and 0.01 probability levels, respectively. Graph was created using Origin software version 2021 (Origin Lab Ltd., Guangzhou, China). Origin is used herein under license. Copyright OriginLab Corporation. All rights reserved. For more information about Origin software, please visit www.originlab.com.

Figure 5 shows that GY was highly significantly (*P* < 0.01) positively correlated with EL, ED, EN and KW, while it was negatively correlated with DM, but the correlation was not significant (*P* > 0.05). There was a significant (*P* < 0.05) positive correlation between EL, ED, KN and KW, while a significant (*P* < 0.05) negative correlation between EL and DM was observed. There was a highly significant (*P* < 0.01) positive correlation between ED, KN and KW, and KN had a highly significant (*P* < 0.01) positive correlation with KW. In short, GY and yield components primarily had a significant (*P* < 0.05) positive correlation. Except for the significant (*P* < 0.05) negative correlation between EL and DM, the correlation between other parameters and DM was weak.

### Ammonia emissions

**Figure 6:**
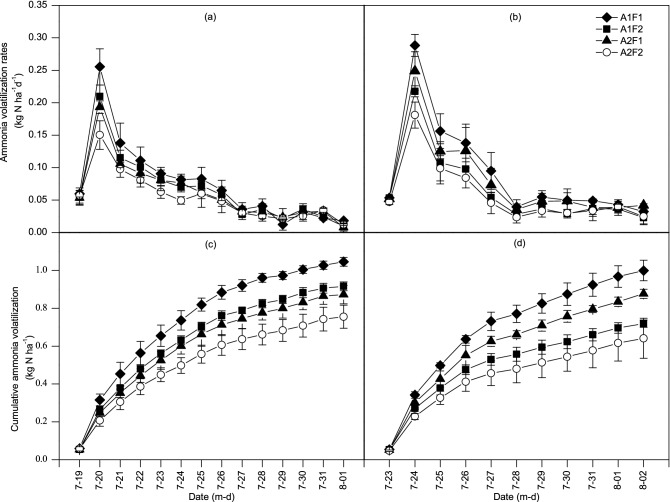
Ammonia volatilization rates as influenced by dripline spacing and fertilization rate treatments of summer maize at the seven-leaf stage in 2017 (**a**) and 2018 (**b**). Cumulative ammonia volatilization as influenced by dripline spacing and fertilization rate treatments of summer maize at the seven-leaf stage in 2017 (**c**) and 2018 (**d**). See Fig. 2 and Table 2 for treatment description. Graphs were created using Origin software version 2021 (Origin Lab Ltd., Guangzhou, China). Origin is used herein under license. Copyright OriginLab Corporation. All rights reserved. For more information about Origin software, please visit www.originlab.com.

The general pattern of ammonia volatilization over time was similar for different dripline spacings and fertilization rates. Ammonia volatilization sharply increased at first and then gradually decreased. The maximum ammonia loss occurred on the first day after fertilization in each treatment. The maximum loss rates of the A1F1, A1F2, A2F1 and A2F2 treatments in 2017 were 0.255, 0.210, 0.193 and 0.150 kg N ha^−1^ day^−1^, respectively, and those in 2018 were 0.288, 0.217, 0.249 and 0.181 kg N ha^−1^ day^−1^. The ammonia volatilization rate gradually decreased from the second day after fertilization until it reached a steady state with small fluctuations. The results for the two-year study showed that the maximum ammonia volatilization rate of the A2 and F2 treatments was reduced by 20.6% and 22.8% compared to those of the A1 and F1 treatments, respectively, indicating that the ammonia volatilization rate increased with increasing fertilization rate and was influenced by dripline spacing. The maximum ammonia volatilization rate occurred in A1F1 group (one drip line per row, high fertilization rate) in both years (Fig. 6a,b), and the average maximum ammonia volatilization rate in the treatment was 0.272 kg N ha^−1^ day^−1^. The maximum ammonia volatilization rate decreased by 21.2%, 19.0%, and 39.2% in the A1F2, A2F1 and A2F2 treatments, respectively, compared with that in the A1F1 treatment.

Cumulative ammonia volatilization in each treatment increased daily throughout the study period in both years, but the rate of increase declined over time (Fig. 6c,d). The order of cumulative ammonia volatilization of the treatments in 2017 was A1F1 > A1F2 > A2F1 > A2F2, and in 2018, it was A1F1 > A2F1 > A1F2 > A2F2. Two-way ANOVA showed that both dripline spacing and fertilization rate significantly affected cumulative ammonia volatilization in 2017 and 2018 and that there was no significant interaction between the two treatments. Ten days after fertigation, the average cumulative ammonia volatilization of the two seasons in 2017 and 2018 from one drip line per two rows treatment was 15.1% lower than that from one drip line per row treatment, and the cumulative volatilization at the low fertilization rate was 20.6% lower than that at the high fertilization rate. The A1F1 treatment had the highest cumulative ammonia volatilization, with an average of 0.986 kg N ha^−1^. The cumulative ammonia volatilization of the A1F2, A2F1 and A2F2 treatments was reduced by 20.5%, 15.0%, and 32.8%, respectively, compared with that of the A1F1 treatment. The loss rate decreased with increasing N fertilization rate, while cumulative ammonia volatilization increased.

### Spatial variability in soil nutrients

**Figure 7:**
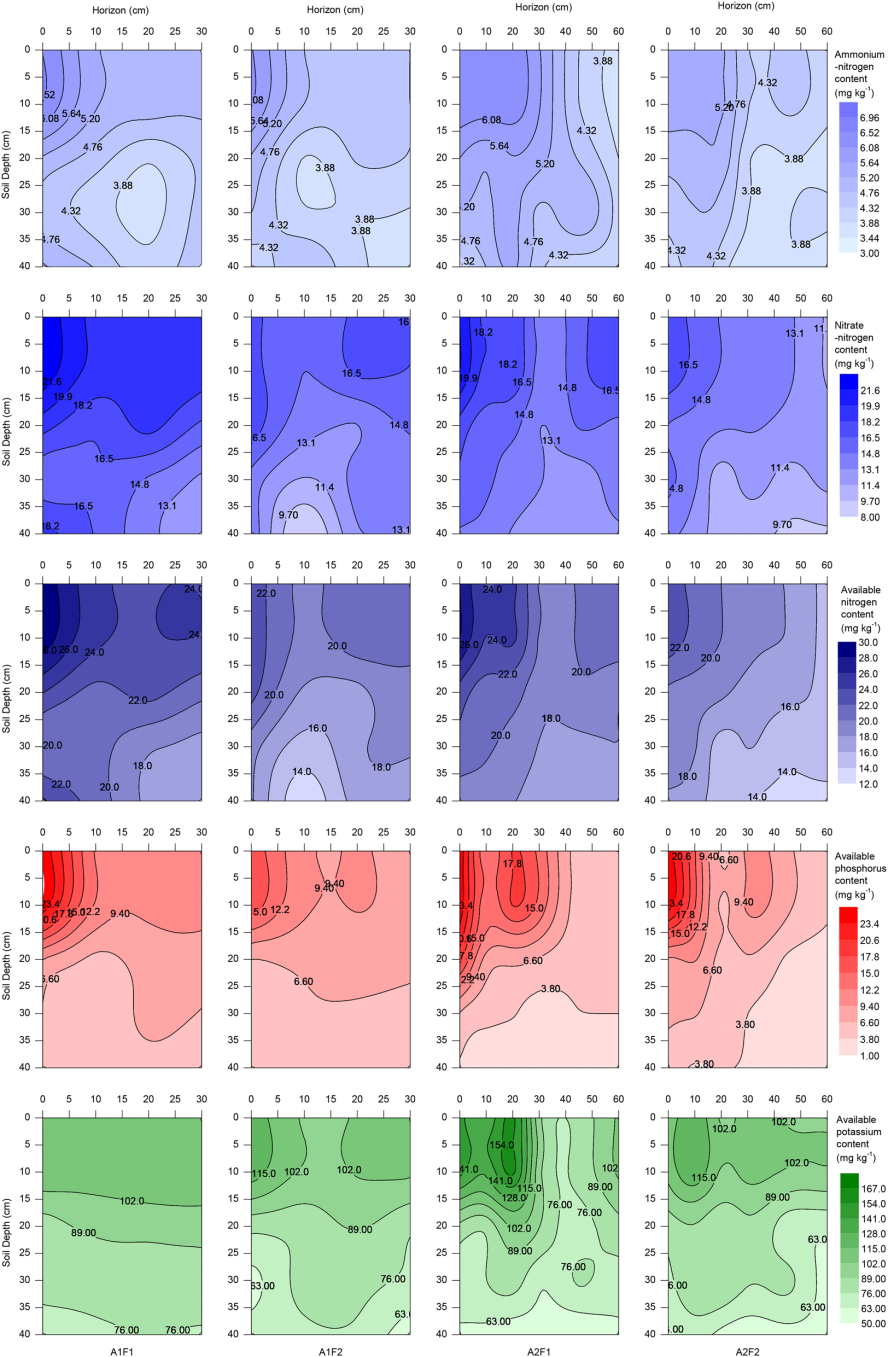
The distribution of soil nutrients content (ammonium-nitrogen, nitrate-nitrogen, available nitrogen, available phosphorus, and available potassium) at harvest in the 2017 maize season. See Fig. 2 and Table 2 for treatment description. Graphs were created using Origin software version 2021 (Origin Lab Ltd., Guangzhou, China). Origin is used herein under license. Copyright OriginLab Corporation. All rights reserved. For more information about Origin software, please visit www.originlab.com.

Soil ammonium-N, available P and available K were concentrated in the topsoil, with a significantly higher content in the 0–10 cm layer than in the lower layers, while nitrate–N was more dispersed in the soil profile due to strong mobility (Fig. 7). The nutrient content of each treatment was significantly (*P* < 0.05) affected by soil depth. The available N and available P contents of each treatment were significantly (*P* < 0.05) affected by horizontal distance. The available K of the A1F1 and A1F2 groups was not affected by the horizontal distance, and the distribution was relatively uniform, while the available K of the A2F1 and A2F2 groups was highly significantly (*P* < 0.01) affected by the horizontal distance (data shown in the appendix).

Dripline spacing had no significant effect on the content of soil available N and K at the maize harvest, but the content of soil available P under the A1 treatment was significantly (*P* < 0.05) higher than that under the A2 treatment (Table 7). Dripline spacing had no significant effect on the CU of soil N, had a highly significant (*P* < 0.01) effect on the CU of soil P, and had a significant (*P* < 0.05) effect on the CU of soil available K. The uniformity of P and K distribution in the A1 treatment was higher than that in the A2 treatment. The high fertilization rate significantly (*P* < 0.05) increased the soil nitrate–N and ammonium-N contents, and highly significantly (*P* < 0.01) increased the soil available N and P contents, but the fertilization rate had no significant effect on the distribution of soil nutrients.

## Discussion

Previous studies have shown that different drip fertigation methods are suitable for different crops in different regions^49–51^. The present study clearly showed that dripline spacing at one line per two rows increased maize yield and N, P and K plant uptake by 10.2%, 7.0%, 8.1% and 1.6%, respectively, compared with one drip line per row (Table 4 and Fig. 4). We originally assumed that the fertilizer applied in the one drip line per row treatment was closer to the roots of maize than in the one drip line per two rows treatment, which would be more conducive to the absorption of fertilizer by maize. However, the results showed that the one drip line per two rows treatment had higher maize yield than the one drip line per row treatment, which was unexpected but reasonable. The reason for the results was complex, one of which could be that the nutrient was too close to the root system and the unsuitable soil nutrient segregation concentration suppressed root growth, the other of which may be that the maize root system could sense nutrients within a certain distance and stimulated root growth to absorb enough nutrients for its own use. It has been reported in the literature that different arrangements of drip lines affect nutrient distribution in the soil and the growth of maize roots and photosynthesis, thereby affecting the absorption and utilization of nutrients by summer maize^52^. Based on this study, it can be concluded that fertigation with one drip line per two rows of maize (i.e., dripline spacing was 1.2 m) was an economical and productive method of drip fertigation, saving approximately half the number of drip lines and obtaining higher yield and nutrient uptake compared to that of one drip line per row of maize, which was similar to the result observed by Bozkurt et al.^15^. We found that the optimum dripline spacing for maize is 1.2 m in sandy loam soil, while Lamm et al.^14^ advocated dripline spacing of 1.5 m obtained the highest yield, highest water use efficiency, and lowest interannual variability in the silt loam soil. It should be noted that Lamm’s study used subsurface driplines installed at a depth of 40–45 cm parallel to the direction of the maize rows. In another study, researchers observed that there was no significant effect of lateral spacings of 60 cm, 75 cm and 90 cm on wheat GY in semihumid areas^16^. The explanation for the results might be that optimum dripline spacing was influenced not only by the crop and its ability to sequester soil nutrients and water in the root zone but also by the soil texture, soil layering and offsite environment^53^. Therefore, further research is needed to evaluate for different crops, soil types, climatic conditions and irrigation availability for their wider applications.

Our study further showed that the fertilization rate in drip fertigation significantly affected the N, P, and K absorption and yield of summer maize, which is similar to the results of previous studies^54,55^, in which combined drip irrigation and fertigation significantly increased the growth of plants. The high fertilization rate in this study increased N, P and K uptake by 23.8%, 20.4% and 14.1%, respectively, compared to the low fertilization rate. The high fertilization rate also significantly (*P* < 0.01) increased the KN. Thus, increasing the rate of N, P and K fertilization in this experiment led to increased absorption of nutrients and yield. Drip fertigation of maize is technically viable in northern China. Given the importance of maize in the region, it is advisable to continue with this line of research; determine the mechanism of increasing yield, physiological changes in maize, and nutrient distribution in the soil; conduct multipoint tests on large plots of land; and improve the economic viability of this system.

The volatilization of ammonia from farmland is a major source of N emissions, which reduces NUE and causes environmental pollution. Research on mitigating ammonia loss has provided important insights for designing effective mitigation strategies targeting different agroecological zones^56–58^. A simple drip irrigation system is easy to structure and can achieve the same effect as deep application of urea in managing ammonia loss^59^. The results of this study showed that manipulation of both dripline spacing and the rate of fertilization could be used to reduce the amount of ammonia volatilization in the summer maize season.

The experimental results in this research demonstrated that the ammonia volatilization rate peaked on the first day after drip fertigation at the summer maize V7 stage. It has been reported that the ammonia volatilization rate reached a maximum on the 2nd to 3rd days after conventional fertilization using different types of urea in the same region^46^. The ammonia volatilization rate reached a maximum quickly in approximately one day in this experiment, which might be attributed to the fact that urea was dissolved in the irrigation water and that hydrolysis of urea occurred more quickly and therefore was able to directly interact with urease in the soil, which agrees with the result observed by Li et al.^60^, who reported that the rate of ammonia volatilization in maize fields increased after fertilization with drip irrigation and reached a maximum on the second day. This study found that the rate of ammonia volatilization slowed two days after drip fertigation, followed by a steady state with small fluctuations for several days, which agrees with previous research results^46^. The results of this research indicated that dripline spacing of one drip line per two rows of maize reduced the ammonia volatilization rate by 20.6% compared to one drip line per row, which may be attributed to the difference in contact area between fertilizer and soil. The finding in this study that reducing the fertilization rate decreased the maximum ammonia volatilization rate by 22.8% is similar to the findings of two previous studies^61,62^, where N fertilizer reduction reduced soil ammonia volatilization.

This study revealed that the order of cumulative ammonia volatilization in 2017 was A1F1 > A1F2 > A2F1 > A2F2, while it was A1F1 > A2F1 > A1F2 > A2F2 in 2018 (Fig. 6). The inconsistent ordering of A1F2 and A2F1 between the two years may be related to some environmental factors, such as temperature^63^, wind speed, solar radiation^64^, and humidity^32^. At the V7 stage, the two-year average cumulative ammonia volatilization from the A1F1, A1F2, A2F1 and A2F2 treatments accounted for 2.2%, 2.5%, 1.9% and 2.1% of the applied N fertilizer, respectively, during this period (Table 6). However, a study by Zhou et al.^46^ showed that the ratio of ammonia volatilization from conventionally applied common urea fertilizer was 6.2–7.4%, and the proportion for controlled release urea and resin coated urea ranged from 4.3 to 5.8%. The above findings indicate that fertilizer application by drip fertigation can reduce the loss of ammonia volatilization from soil compared to conventional fertilization, which somewhat conflicts with other reports^32^. The explanation for this result was complex, and different fertigation systems, crops and fertilizer regimes require further research.

**Table 6:** Cumulative ammonia volatilization and its proportion of nitrogen fertilization rate under different treatments of summer maize at the seven-leaf stage in 2017 and 2018.

Year	Treatment^a^	Nitrogen fertilization rate (kg N ha^−1^)	10-day cumulative ammonia loss (kg N ha^−1^)	Loss rate (%)	Decrease in ammonia loss relative to A1F1 (%)
2017	A1F1	45	0.97a^b^	2.16b	–
	A1F2	31.5	0.85b	2.69a	13.00
	A2F1	45	0.80b	1.78c	17.77
	A2F2	31.5	0.68c	2.17b	29.81
2018	A1F1	45	1.00a	2.22a	–
	A1F2	31.5	0.72c	2.28a	28.08
	A2F1	45	0.88b	1.95a	12.30
	A2F2	31.5	0.64c	2.04a	35.81
2017	Source of variance		*F* value	*F* value	
	A		49.35**	46.02**	
	F		25.78**	47.32**	
	A × F		0.04	1.03	
2018	Source of variance		*F* value	*F* value	
	A		7.96*	6.01*	
	F		52.60**	0.50	
	A × F		0.41	0.02	

^a^A and F represent dripline spacing and fertilization rate, respectively, as shown in Fig. 2 and Table 2.

^b^Values within a column followed by different letters are significantly different at the 0.05 probability level; * and ** show significant difference at the 0.05 and 0.01 probability levels, respectively.

Usually, maize yield and nutrient uptake have a close relationship with the distribution of soil nutrients. The amount of fertilization and irrigation under the A1 and A2 groups were the same, and they could be controlled by the drip fertigation system. As the number of driplines under A1 was twice that under A2, they required different times for fertilization. Dripline spacing affected the infiltration and redistribution of water, which could further affect the spatial variability in nutrients. The contents of ammonium-N, P and K in the topsoil were higher than in greater soil depths (Fig. 7) because they were positively charged, while the soil colloids were negatively charged, and they were more easily adsorbed by the soil colloids, resulting in poor mobility. In our study, the spatial variability in soil P and K levels were significantly (*P* < 0.01) influenced by dripline spacing, and the distribution of soil P and K of one drip line per row was more even, so the CV was smaller (Table 7). While a smaller CV did not mean a higher yield, the explanation for the results could be that the suitable high variability in soil nutrients or nutrient stress, similar to drought stress, led to compensation effects in plants^65^. The high fertilization rate significantly (*P* < 0.05) increased the content of ammonia-N, nitrate–N, available N, and available P but only significantly (*P* < 0.05) increased the CV of ammonia-N. The reason for this observation might be that ammonia-N in the soil nutrient pool was easily oxidized, resulting in a lower N content and therefore increased plant sensitivity to fertilization. This research revealed the spatial variability in soil nutrients under different dripline spacings and fertilization rates, which can help us explore suitable methods for managing soil nutrients, recommend fertilization and improve soil quality. It could be concluded that dripline spacing affected the concentration and distribution of soil nutrients, managed ammonia volatilization losses, and affected plant growth and yield formation. This study provided a research direction for coordinating the relationship between nutrient concentration and irrigation efficiency to increase GY.

**Table 7:** Effects of dripline spacing and fertilization rate on soil nutrient parameters at harvest in the 2017 maize season.

Treatment	Ammonium-nitrogen content			Nitrate-nitrogen content			Available nitrogen content			Available phosphorus content			Available potassium content		
	AV (mg kg^−1^)^a^	CV (%)	CU (%)	AV (mg kg^−1^)	CV (%)	CU (%)	AV (mg kg^−1^)	CV (%)	CU (%)	AV (mg kg^−1^)	CV (%)	CU (%)	AV (mg kg^-1^)	CV (%)	CU (%)
A1F1^b^	4.71ab^c^	17.60a	84.38a	16.94a	20.02a	82.12a	21.65a	18.10a	84.77a	8.31a	61.39bc	61.61ab	88.99a	19.57c	83.34a
A1F2	4.38b	15.54a	86.57a	14.13b	18.39a	83.81a	18.51bc	15.70a	86.99a	7.16b	44.23c	69.54a	82.40ab	22.51bc	82.57a
A2F1	4.97a	17.81a	85.36a	15.01ab	16.00a	87.69a	19.98ab	14.49a	88.83a	7.27b	85.32a	36.74c	83.49ab	33.98a	74.97b
A2F2	4.36b	15.49a	86.80a	12.77b	17.86a	85.61a	17.13c	16.25a	86.85a	6.40c	79.15ab	47.21bc	80.06b	25.05b	79.51ab
Source of variance	*F* value	*F* value	*F* value	*F* value	*F* value	*F* value	*F* value	*F* value	*F* value	*F* value	*F* value	*F* value	*F* value	*F* value	*F* value
A	0.65	0.02	0.25	5.4	0.55	4.46	6.92	0.41	1.22	46.10**	29.78**	28.56**	4.24	42.58**	20.94*
F	10.23*	12.10*	2.25	12.65*	0	0.01	26.56**	0.02	0	58.35**	4.68	4.34	6.92	5.32	2.27
A*F	0.98	0.04	0.1	0.17	0.32	1.17	0.06	0.77	1.4	1.11	1.04	0.08	0.69	20.86*	4.51

^a^AV is the average soil nutrient content, CV is the coefficient of variation, and CU is the Christiansen uniformity coefficient.

^b^A and F represent dripline spacing and fertilization rate, respectively, as shown in Fig. 2 and Table 2.

^c^Values within a column followed by different letters are significantly different at the 0.05 probability level; * and ** show significant difference at the 0.05 and 0.01 probability levels, respectively.

The North China Plain belongs to the northern temperate monsoon climate zone, which is characterized by a cold and dry winter and hot and wet summer and has four distinct seasons. Seasonal drought, low water and fertilizer utilization efficiencies and a shortage of fresh water are major factors limiting agricultural production in this region. A preliminary study was carried out to investigate the effects of fertilizer under different dripline spacings on summer maize yield in northern China. The findings from this study and those from the literature clearly indicated that drip fertigation might not have a large environmental impact, could reduce fertilization labour input and could significantly improve fertilizer use efficiency in this region^66^. Our study demonstrated that drip fertigation in intensive cropping systems is a potential option for effective fertilization technologies and to cope with improving the absorption and utilization of fertilizer by crops and protecting the environment. Fertigation is promising as a potentially suitable fertilization method for crop producers, which agrees with previous research^32^. However, most of the rainfall was concentrated in the summer maize growing season in both study years, which could basically meet the water requirements of summer maize, so the level of irrigation was low. Therefore, it was difficult to observe the water-saving effects of drip fertigation in summer maize in northern China, which might become a disadvantage of drip fertigation technology.

## Conclusions

The results of this study indicated that maize yield was significantly (*P* < 0.05) affected by both dripline spacing and fertilization rate. The yield of maize in the treatment with one drip line per two rows was, on average, 10.2% higher than that in the treatment with one drip line per row. Maize yield at the low fertilization level (139.5 kg ha^−1^ N, 76.5 kg ha^−1^ P_2_O_5_, 76.5 kg ha^−1^ K_2_O) was 9.8% lower than that at the high fertilization level (180 kg ha^−1^ N, 90 kg ha^−1^ P_2_O_5_, 90 kg ha^−1^ K_2_O). Total N, P, and K uptake by the aboveground part of plants at harvest in the treatment with one drip line per two rows was 7.0%, 8.1% and 1.6% higher than that in the treatment with one drip line per row treatment, respectively, and that in the high fertilization level treatment were 23.8%, 20.4% and 14.1% higher than that in the low fertilization level treatment, respectively. The quantity of cumulative ammonia volatilization in the treatment with one drip line per two rows was reduced by 15.1% compared to the treatment with one drip line per row and that in the low fertilization level treatment decreased by 20.6% compared with that in the high fertilization level treatment. These results indicated that one drip line per two rows of maize increased the GY via increased plant nutrient uptake and reduced ammonia volatilization. The high fertilization rate could increase the maize yield, but the ammonia volatilization loss was higher than at the low fertilization rate. Overall, one drip line per two rows with the high fertilization rate treatment resulted in a higher yield and nutrient uptake than the other treatments, could have a lower environmental impact and is therefore a suitable fertigation system in northern China. Future research needs to be conducted to better understand the underlying mechanisms of fertilization under drip fertigation for designing effective fertigation strategies and increasing crop yield for green agricultural development.

## Supplementary Information

Supplementary Information.
