# Supplementary material for: Effects of fertilizer under different dripline spacings on summer maize in northern China
Source: Sci Rep. 2021 Sep 23;11:18922. doi: 10.1038/s41598-021-98016-6 (PMC8460782; doi:10.1038/s41598-021-98016-6)
Supplement: Supplementary file 1 — Supplementary Information. [file 41598_2021_98016_MOESM1_ESM.pdf]

**Effects of fertilizer under different dripline spacings on summer maize in northern China**

Ge Li[1], Youlu Bai[1,*], Lei Wang[1, **], Yanli Lu[1], Jingjing Zhang[2], Yinjie Zhang[1]

[1] *Key Laboratory of Plant nutrition and Fertilizer, Ministry of Agriculture and Rural Affairs/ Institute of Agricultural Resources and Regional Planning, Chinese Academy of Agricultural Sciences, Beijing, 100081, PR China*

[2] *Institute of Environment and Sustainable Development in Agriculture, Chinese Academy of Agricultural Sciences, Beijing, 100081, PR China*

**The e-mails of all co-authors:**

Ge Li: lige_gege@126.com;

Youlu Bai: baiyoulu@caas.cn;

Lei Wang: wanglei02@caas.cn;

Yanli Lu: luyanli@caas.cn;

Jingjing Zhang: zhangjingjing0827@163.com;

Yinjie Zhang: zzhangyinjie@126.com.

*** Corresponding author:** Youlu Bai.

**** Corresponding author:** Lei Wang.

**Full postal address:** 12 South Road, Zhongguancun, Haidian District, Institute of Agricultural Resources and Regional Planning, Chinese Academy of Agricultural Sciences, Beijing, 100081, China

**E-mail address:** baiyoulu@caas.cn (Y. Bai), wanglei02@caas.cn (L. Wang).

Supplements

Appendix A

**Table A**

Effects of horizontal distance (H) and soil depth (D) on soil nutrients content in each treatment in the 2017 maize season.

| Item | | Ammonium-nitrogen content (mg kg$^{-1}$) | | | | Nitrate-nitrogen content (mg kg$^{-1}$) | | | | Available nitrogen content (mg kg$^{-1}$) | | | | Available phosphorus content (mg kg$^{-1}$) | | | | Available potassium content (mg kg$^{-1}$) | | | |
|---|---|---|---|---|---|---|---|---|---|---|---|---|---|---|---|---|---|---|---|---|---|
| | | A1F1 [a] | A1F2 | A2F1 | A2F2 | A1F1 | A1F2 | A2F1 | A2F2 | A1F1 | A1F2 | A2F1 | A2F2 | A1F1 | A1F2 | A2F1 | A2F2 | A1F1 | A1F2 | A2F1 | A2F2 |
| Horizon (cm) | 0 | 5.37a[b] | 5.01a | 5.34ab | 4.70a | 19.06a | 15.79a | 17.50a | 15.31a | 24.43a | 20.81a | 22.84a | 20.01a | 11.34a | 8.10a | 13.53a | 11.40a | 85.78a | 81.95a | 89.97b | 80.14b |
| | 10 | 4.65ab | 4.32b | 5.36ab | 4.92a | 17.25ab | 11.98b | 15.62b | 13.96b | 21.90ab | 16.29c | 20.98b | 18.89a | 6.90b | 7.22b | 7.30bc | 8.82b | 88.60a | 83.79a | 86.03bc | 91.54a |
| | 20 | 4.18b | 4.21b | 5.65a | 4.77a | 16.42ab | 14.11a | 15.14bc | 12.25cd | 20.61b | 18.32b | 20.80b | 17.02b | 7.68b | 6.95b | 7.95b | 5.26cd | 90.64a | 86.44a | 99.57a | 81.54b |
| | 30 | 4.63ab | 4.00b | 5.02bc | 3.95b | 15.03b | 14.64a | 13.20d | 12.53c | 19.66b | 18.64b | 18.22c | 16.48b | 7.34b | 6.36b | 6.53c | 5.91c | 90.96a | 77.43a | 75.93d | 80.74b |
| | 45 | - | - | 4.58c | 3.99b | - | - | 13.94cd | 11.51cd | - | - | 18.51c | 15.50bc | - | - | 3.90d | 3.90de | - | - | 70.11d | 76.68bc |
| | 60 | - | - | 3.90d | 3.80b | - | - | 14.64bc | 11.07d | - | - | 18.53c | 14.88c | - | - | 4.43d | 3.09e | - | - | 79.30cd | 69.76c |
| Depth (cm) | 0 – 10 | 5.41a | 4.94a | 5.46a | 4.83a | 19.96a | 16.33a | 17.47a | 14.53a | 25.37a | 21.26a | 22.93a | 19.36a | 14.42a | 11.13a | 13.72a | 11.26a | 110.65a | 107.10a | 118.18a | 106.37a |
| | 10–20 | 4.52b | 4.41b | 5.07ab | 4.40b | 17.67ab | 14.85ab | 15.15b | 13.33b | 22.19ab | 19.26b | 20.23b | 17.73b | 7.56b | 7.38b | 8.49b | 6.16b | 90.86b | 82.21b | 85.70b | 78.42b |
| | 20–30 | 4.23b | 4.11b | 4.94b | 4.27bc | 14.86b | 13.41bc | 14.13bc | 12.31b | 19.09b | 17.52bc | 19.07c | 16.58b | 6.31c | 5.67c | 4.22c | 4.73c | 81.45bc | 73.86bc | 73.28c | 76.98b |
| | 30–40 | 4.68ab | 4.07b | 4.43c | 3.92c | 15.27b | 11.94c | 13.26c | 10.92c | 19.95b | 16.01c | 17.69d | 14.84c | 4.77d | 4.46d | 2.66d | 3.44d | 73.01c | 66.43c | 56.79d | 58.49c |
| Source of variance | D | 3.60* | 7.16** | 7.68** | 8.18** | 4.98* | 8.30** | 25.41** | 18.42** | 6.43** | 12.65** | 32.01** | 21.89** | 94.93** | 110.85** | 171.94** | 62.13** | 13.03** | 28.98** | 107.46** | 79.38** |
| | H | 3.40* | 8.71** | 11.52** | 9.27** | 2.53 | 5.98** | 11.43** | 13.25** | 3.50* | 8.44** | 14.78** | 15.70** | 21.95** | 6.89** | 55.79** | 34.95** | 0.28 | 1.33 | 11.90** | 6.84** |
| | H*D | 0.28 | 2.16 | 1.5 | 0.49 | 0.43 | 0.84 | 0.98 | 0.86 | 0.41 | 0.61 | 1.13 | 0.83 | 16.92** | 14.02** | 14.52** | 8.94** | 0.1 | 1.78 | 5.76** | 1.29 |

[a] A and F represent dripline spacing and fertilization rate, respectively, as shown in Fig. 2 and Table 2.

[b] Values within a column followed by different letters are significantly different at the 0.05 probability level; * and ** show significant difference at the 0.05 and 0.01 probability levels, respectively.
